# P-1543. Clinical Characteristics and Factors Associated with Acquisition of Gram-Negative Bacteria during Ceftazidime-avibactam Therapy

**DOI:** 10.1093/ofid/ofae631.1710

**Published:** 2025-01-29

**Authors:** Chien Chuang, Tzu-Chi Kao, Sheng-Hua Chou, Chih-Han Juan, Yu-Chien Ho, Szu-Yu Liu, Yi-Tsung Lin

**Affiliations:** Division of Infectious Diseases, Department of Medicine, Taipei Veterans General Hospital, Taipei, Taiwan, Taipei, Taipei, Taiwan (Republic of China); Taipei Veterans General Hospital, Taipei, Taipei, Taiwan; Institute of Emergency and Critical Care Medicine, National Yang-Ming University, Taipei, Taiwan, Taipei, Taipei, Taiwan (Republic of China); Taipei Veterans General Hospital and National Yang Ming Chiao Tung University, Taipei, Taipei, Taiwan; Taipei Veterans General Hospital, Taipei, Taipei, Taiwan; Taipei Veterans General Hospital, Taipei, Taipei, Taiwan; Taipei Veterans General Hospital and National Yang Ming Chiao Tung University, Taipei, Taipei, Taiwan

## Abstract

**Background:**

Ceftazidime-avibactam (CZA) is recommended to treat infections caused by carbapenem-resistant *Enterobacterales* (CRE) and *Pseudomonas aeruginosa* with difficult-to-treat resistance. The subsequent isolation of gram-negative bacteria (GNB) would cause superinfection or result in the change of antibiotics. However, the clinical characteristics and factors for acquisition of GNB during CZA treatment has not been reported.

Demographic and clinical characteristics of patients who acquired Gram-negative bacteria and those who did not acquire Gram-negative bacteria during ceftazidime-avibactam therapy

**Figure:**
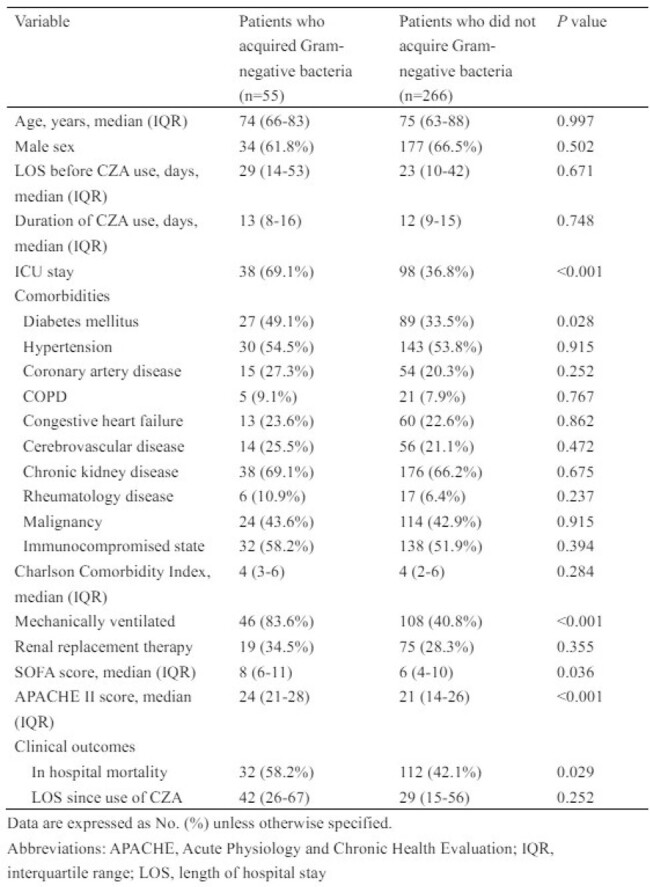

**Methods:**

Adult patients treated with CZA ≥ 5 days were retrospectively enrolled at Taipei Veterans General Hospital (December 2019 to June 2021). We compared clinical features of patients who acquired GNB from clinical specimen 5 days after the use of CZA and those who did not acquire any GNB. Multivariate analysis was used to explore risk factors for acquisition of GNB during and 28-day mortality in patients who acquired GNB.

Distribution of acquisition of Gram-negative bacteria during ceftazidime-avibactam therapy

**Figure:**
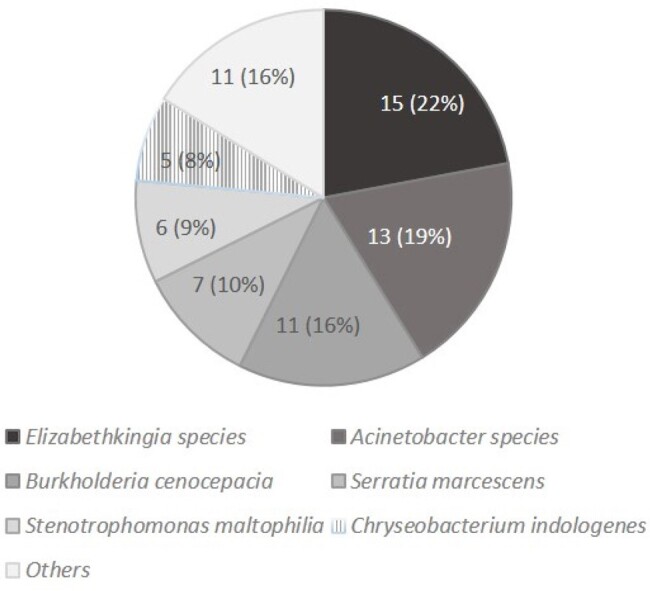

**Results:**

A total of 321 patients receiving CZA for ≥ 5 days was analyzed, and 67.3% of them were infected with CRE. A total of 68 GNB were identified from the 55 patients (17.1%, 55/321). *Elizabethkingia* species (n=15) was the most common species, followed by *Acinetobacter* species (n=13) and *Burkholderia cenocepacia* (n=11). Diabetes mellitus [odds ratio (OR), 2.12; P=0.022], and use of mechanical ventilation (OR, 6.47; P=0.001) were the independent risk factors for acquisition of GNB in the multivariate analysis. In hospital mortality in patients who acquired GNB was higher than those who did not acquire any GNB during CZA treatment (58.2% versus 42.1%, P=0.029). More than half (63.6%, 35/55) of patients acquiring GNB were considered to have superinfection, and antibiotics were changed. Cerebrovascular disease [hazard ratio (HR), 3.27; P=0.009] and SOFA score at acquisition (HR, 1.22; P=0.001) were associated with increased 28-day mortality in patients who acquired GNB.

**Conclusion:**

*Elizabethkingia* species, *Acinetobacter* species, and *Burkholderia cenocepacia* were the major GNB identified during CZA treatment, and patients who had diabetes mellitus and use of mechanical ventilation were at higher risk of acquisition of GNB. Patients who acquired GNB had higher in-hospital mortality rate. Cerebrovascular disease and disease severity were determinants of 28-day mortality.

**Disclosures:**

**All Authors**: No reported disclosures

